# Unlocking the potential of exosomes: A new frontier in liver cancer liquid biopsy

**DOI:** 10.1016/j.jlb.2024.100166

**Published:** 2024-09-02

**Authors:** Asmit Das, Sidhanti Nyahatkar, Swarup Sonar, Ketki Kalele, Vetriselvan Subramaniyan

**Affiliations:** a Department of Oncology, Neuron Institute of Applied Research, Amravati, Maharashtra, India; b Department of Dentistry, VYWS Dental College & Hospital, Amravati, Maharashtra, India; c Center for Global Health Research, Saveetha Medical College, Saveetha Institute of Medical and Technical Sciences, India; d Department of Medical Sciences, School of Medical and Life Sciences, Sunway University, Bandar Sunway, 47500, Subang Jaya, Selangor, Malaysia

**Keywords:** Extracellular vesicle, Exosomes, Liver cancer, Metastasis, Liquid biopsy, Biomarkers

## Abstract

Liquid biopsy has emerged as one of the non-invasive diagnostic strategies for cancer, offering significant advantages over traditional tissue biopsies. Exosomes the nanoscale extracellular vesicles, have significantly been in the spotlight of research and investigation as highly informative biomarkers in liquid biopsy. These vesicles, which are secreted by a variety of cells, including tumor cells, contain useful information on the molecular characteristics of the parent cell and could be used as a mirror into the processes underlying cancer biology. The analysis of the biomolecular exosomal cargo, including proteins, nucleic acids, and lipids, has shown great promise for the development of sensitive and specific liquid biopsy-based biomarkers for cancer detection, monitoring, and prognosis. This review discusses the role of exosomes in the liver cancer development and metastatic process, including their ability to transfer oncogenic material and facilitate tumor progression. It also explores the application of exosomes as a tool for early cancer detection, monitoring disease status, and predicting prognosis, with a specific focus on liver cancer. Exosomes hold great promise as a minimally invasive liquid biopsy approach that could revolutionize the way we diagnose and manage this deadly disease.

## 1 Introduction

**Fig. 1:**
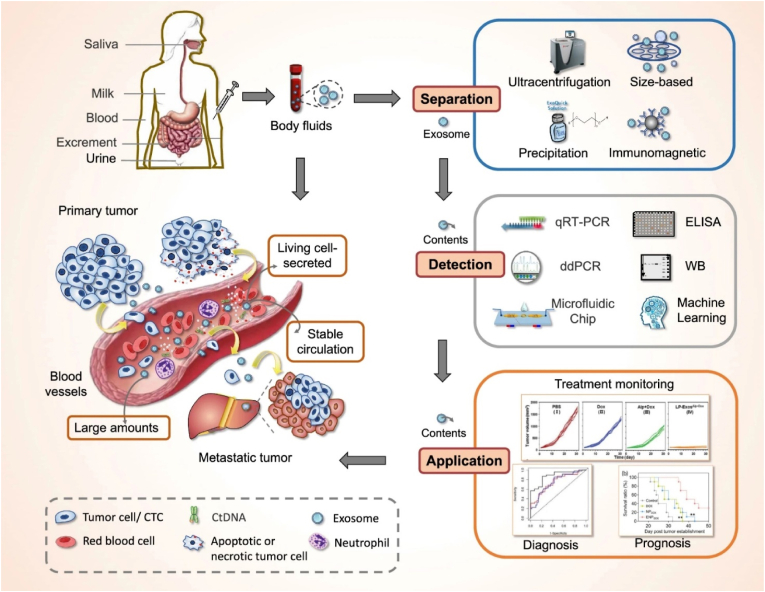
Exosomes-based Liquid biopsy approach. (Reproduced with permission under Creative Commons CC BY 4.0 license from Ref. [17] Copyright @ 2022 The Authors).

Primary malignancy of hepatocytes poses a significant global health burden, it belongs to top 10 cancer-associated health complications [1,2]. This is underscored by the fact that liver cancer has a higher frequency of recurrence, owing to its high mortality rates. This is often attributed to late diagnosis, motivating researchers to develop the most efficient experimental methods [3]. The current clinical diagnosis of hepatocellular carcinoma relies on a multi-pronged approach. This includes assessing serum present α-fetoprotein, ultrasound and imaging method (MRI). In certain cases, an invasive tumor biopsy may also be necessary [4]. While traditional diagnostic approaches like imaging and tissue biopsies are valuable, they can be invasive, prone to sampling bias, and may not fully capture the complex heterogeneity of the tumor. Liquid biopsy approaches are based on circulating tumor cells, circulating biomolecules (DNA, RNA, etc.), and exosome profiling [5]. Exosomes are a subpopulation of EVs and originate from endosomes [6]. In cancer research, exosomes have a significant role in cancer liquid biopsy (Fig. 1) and therapeutic development [7,8,9]. Exosomes-based hepatocellular carcinoma (HCC) profiling is an impressive diagnostic and prognostic approach [10]. Several biofluids such as blood, plasma, serum, urine and ascites have been used in liquid biopsy [11]. CTCs and exosomes, shed from the primary tumor into the bloodstream, provide valuable insights into the metastatic potential of the tumor [12]. Exosomes are nanoscale extracellular vesicles that are secreted by a variety of cells, including cancer cells. These cellular communicating nanovesicles also play a significant role in biomarker research. During cancer development, it regulates several events such as uncontrolled cell growth, immunosuppression, angiogenesis, metastasis, and drug and therapeutic resistance development. Tumor-derived exosomes (TEXs) based VEGF expression promotes angiogenesis and lead to liver metastasis [13]. Exosomal integrins, which act as "zip codes" for metastasis, can dictate the organ-specific spread of cancer cells with certain types linked to liver metastasis [14]. Exosomal miRNAs is implicated in tumor growth, metastasis, and drug resistance [15]. Imagine a future where a simple body fluid test could unlock a deeper understanding of liver cancer, paving the way for earlier detection, tailored treatments, and ultimately, better outcomes for patients. In the exosomes clinal trial, a large number of cancer biomarker-based trials (almost 80 %) research indicate that exosomes become more promising in cancer detection [16]. This is the promise held by exosomes, that carry a treasure trove of information about the inner workings of tumors. This article explores the fascinating world of exosomes, exploring their origins, their diverse cargo, and their emerging role as powerful liquid biopsy biomarkers in the fight against liver cancer.

## 2 Exosome biogenesis

**Fig. 2:**
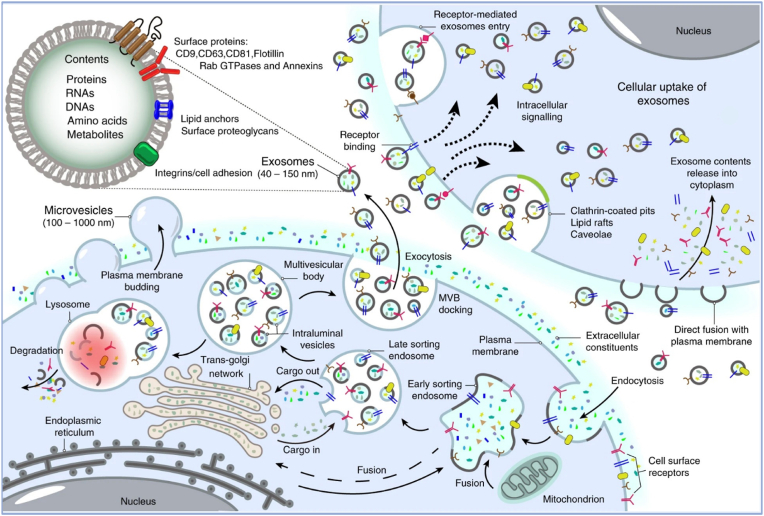
Exosomes biogenesis (Reproduced with permission under Creative Commons CC BY 4.0 license from Ref. [25] Copyright @2020 Nature).

Exosomes biogenesis (Fig. 2) is a complex process of endosome maturation [6]. It transports several bioactive molecules such as DNA, RNA, proteins, and lipids [18]. These molecular cargos carry cancer biomarkers. The endosomal maturation process stapes are early endosomes into late endosomes, and later it develop into microvesicular bodies (MVBs) [19]. MVBs utilize the Endosomal Sorting Complex Required for Transport (ESCRT) to sort cargo and deform membranes. ESCRT complex supports the fusion of MVBs with the plasma membrane and releases exosomes [20]. ESCRT 0 to III has a significant role in exosome biogenesis [21,22]. ESCRT-independent exosome biogenesis mechanisms are regulated via tetraspanin [23] (this protein is involved in several cancer development stages) and several lipids. Tetraspanins like CD9, CD63, and CD82 are crucial, with CD82 aiding cargo sorting and CD9 depletion reducing exosome release. Lipids also play a role in exosome biogenesis. The hydrolysis reaction of sphingomyelinases converts sphingomyelin into ceramide. This ceramide supports MVBs to fuse with the plasma membrane and release exosomes. ESCRT-independent exosome biogenesis is a major pathway of tumor exosome release [24].

## 3 Role of exosomes in liver cancer

**Fig. 3:**
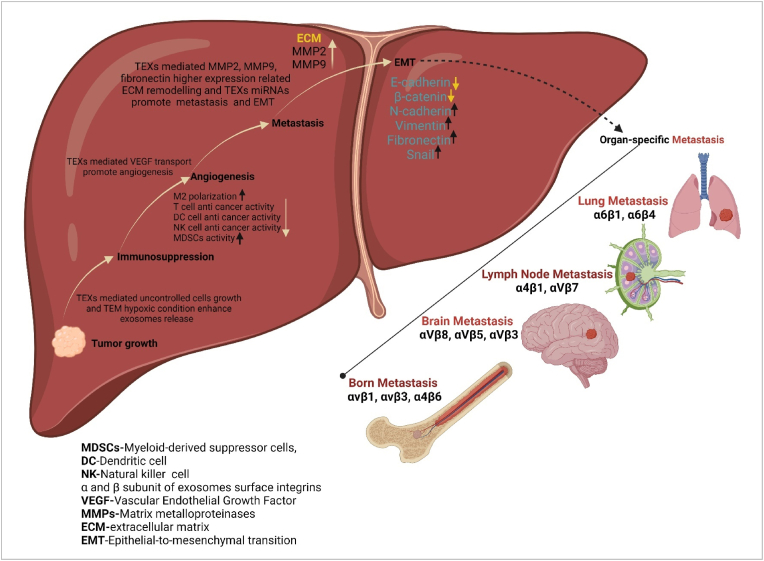
Role of exosomes in liver cancer (created with biorender).

Exosomes are signalling molecules in the tumor microenvironment. Tumor-derived exosomes (TEXs) mediated molecular cargo transport has grate impact in tumor growth, metastasis, and immune evasion. Liver cancer cells release exosomes to suppress anti-tumor immunity through multifaceted mechanisms, creating an immunosuppressive microenvironment that facilitates their survival and proliferation. Tumor-derived exosomes can polarize macrophages towards an M2 phenotype, induce dendritic cell dysfunction, and directly suppress T cell function, leading to immune evasion. For instance, exosomes carrying miRNA-21 and miRNA-23a-3p promote M2 polarization [26,27], exosomes with TGF-β suppress dendritic cell maturation [28], and exosomal PD-L1 binds to PD-1 on T cells [29], ultimately inhibiting anti-tumor immune responses. Exosomal microRNAs (miRNAs) have emerged as promising biomarkers for liver cancer, offering the potential for non-invasive disease monitoring and management. Exhibiting tumor-specific expression patterns and stability in biofluids, exosomal miRNAs hold immense value for diagnostic, prognostic, and therapeutic applications. Several studies have identified differentially expressed exosomal miRNAs in liver cancer patients compared to healthy individuals, highlighting their utility as diagnostic biomarkers. For instance, elevated levels of serum exosomal miRNA-21 have demonstrated high sensitivity and specificity for hepatocellular carcinoma (HCC) detection [26]. In HCC patient exosomes miRNA-224 expression pattern indicates the complication of the problem [30]. Overall, the multifaceted roles of exosomal miRNAs in liver cancer underscore their significance as non-invasive biomarkers and therapeutic targets [31]. Exosomes play a critical role in promoting angiogenesis, the formation of new blood vessels, and metastasis, the spread of cancer cells to distant organs - both hallmarks of aggressive liver cancer. Tumor-derived exosomes can stimulate angiogenesis by delivering pro-angiogenic factors to endothelial cells, the building blocks of blood vessels, with exosomal miRNA-155 promoting angiogenesis by targeting VHL, a negative regulator, leading to increased vascularization and tumor growth [32]. Exosomes contribute to metastasis by preparing distant organs for tumor cell arrival, a process known as pre-metastatic niche formation. For example, exosomes from liver cancer cells can "educate" bone marrow-derived cells, promoting their mobilization to the lung and creating a favorable environment for metastatic colonization [33]. Exosomal miRNA-122 has also been shown to promote HCC metastasis by enhancing tumor cell migration and invasion [4]. Together, these findings highlight the critical role of exosomes in driving angiogenesis and metastasis, two hallmarks of aggressive liver cancer. Exosomes play a crucial role in the epithelial-to-mesenchymal transition (EMT) [34], a critical process in cancer progression that enables epithelial tumor cells to acquire more motile and invasive mesenchymal traits, thereby facilitating metastasis. Emerging evidence suggests that exosomes can influence organ-specific metastasis, partially mediated by interferons, a family of cytokines with antiviral and immunomodulatory functions. Studies have shown that IFN-α treatment can induce the release of exosomes from tumor cells, which can then "prime" distant organs for metastasis by altering the extracellular matrix composition and immune cell profiles in target organs, creating a more receptive environment for tumor cell colonization [35]. Additionally, the specific cargo of exosomes, including miRNAs and proteins, may dictate their organotropism, influencing which organs are preferentially targeted for metastasis, with exosomes enriched with specific integrins directing metastasis to specific organs [5]. TEXs play a dynamic role in HCC progression (Fig. 3), orchestrating immune suppression, angiogenesis, metastasis, and even influencing organ-specific dissemination. Their ability to transfer bioactive molecules, particularly miRNAs, between cells makes them powerful mediators of intercellular communication within the tumor microenvironment. Understanding the intricate mechanisms by which exosomes contribute to liver cancer pathogenesis is crucial for developing novel diagnostic and therapeutic strategies. Targeting exosomes or their cargo holds immense promise for disrupting tumor progression, enhancing anti-tumor immunity, and ultimately improving patient outcomes.

## 4 Exosome-based liquid biopsy for liver cancer

Exosome-based liquid biopsy is a promising approach for the detection of liver cancer. Human biofluid-based exosome screening for liver cancer (Table 1). In the clinical trials, most trials focus on biomarker research (Table 1).

**Table 1:** Exosome-based biomarkers and clinical trials for liver cancer.

Biomarkers	Exosomes source	Exosome cargo	Clinical importance	References
**Diagnostic**	Plasma	miRNA-106b-5p	miRNA-106b-5p overexpression may promote HCC cell proliferation and invasion by suppressing FOG2, implying its upregulation may promote the aggressive progression of HCC.	[36]
	Serum	miRNA-21	Elevated levels are associated with early HCC diagnosis, Maybe used as a prognostic marker at advanced stages.	[26]
**Prognostic**	Plasma	PD-L1	High exosomal PD-L1 levels predict poor response to immunotherapy and worse survival in various cancers, including HCC.	[37]
		lncRNA-H19	High plasma exosomal lncRNA-H19 levels were significantly associated with poor overall survival in HCC patients. They suggest that lncRNA-H19 may promote HCC progression by sponging miRNA-520a-3p.	[38]
		Alpha-fetoprotein mRNA	Exosomal AFP mRNA levels were significantly higher in HCC patients compared to controls and correlated with tumor stage and metastasis.	[39]
	Serum	miRNA-224	High levels correlate with advanced tumor stage, metastasis, and poor survival in HCC.	[30]

Clinical trial			
Clinical trial ID	Exosomes source	Clinical importance	Funding
NCT06342414	Blood	An Exosome-Based Liquid Biopsy for the Differential Diagnosis of Primary Liver Cancer	City of Hope Medical Center
NCT05375604	exoASO-STAT6 (CDK-004)	This is a first-in-human, Phase 1 open-label, multicenter, dose escalation, safety, pharmacodynamic, and PK study of exoASO-STAT6 (CDK-004) in patients with advanced Hepatocellular Carcinoma (HCC) and patients with liver metastases from either primary gastric cancer or colorectal cancer (CRC).	Codiak Bio Sciences
NCT05575622	Circulating exosomes	Clinical Study for Combined Analysis of CTC and Exosomes on Predicting the Efficacy of Immunotherapy in Patients with Hepatocellular Carcinoma	Zhongnan Hospital
NCT06381648	Not mentioned	Detecting Lymph Node Metastasis in Intrahepatic Cholangiocarcinoma	City of Hope Medical Center

## 5 Challenges and future orientation

**Fig. 4:**
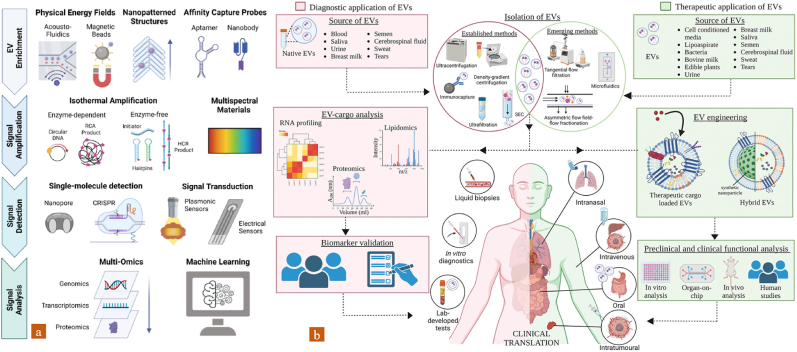
Exosomes profiling to transpersonal research. a)single EV profiling (Reproduced with permission from Ref. [42] Copyright @ 2022 American Chemical Society), b) Exosomes based transpersonal research (Reproduced with permission under Creative Commons CC BY 4.0 license from Ref. [44] Copyright @ 2023 The Authors).

Exosome-based liquid biopsy holds immense promise for revolutionizing liver cancer management, but several challenges must be addressed before its widespread clinical implementation [40]. Exosome isolation complication, heterogeneity, and molecular diversity are major concerns in exosomes-based liquid biopsy [41]. Furthermore, the inherent heterogeneity of exosomes, is influenced by factors like tumor stages based exosome size variation, origin (normal/tumor cell), and molecular diversity [42]. The future of exosome-based liquid biopsy in liver cancer management is bright, fueled by ongoing research and technological advancements poised to enhance isolation, analysis, and clinical application. Single exosome profiling (Fig. 4), exosome barcoding, multi-omics profiling [43], Microfluidic devices and nanotechnology offer improved exosome isolation and analysis, enabling high-purity extraction and sensitive biomarker detection [6]. Simultaneously, single-molecule detection techniques promise to unravel exosome heterogeneity and uncover rare biomarkers. Ultimately, exosomes hold immense potential for advancing personalized medicine, enabling real-time monitoring of tumor evolution and treatment response, guiding personalized therapeutic interventions, and even serving as engineered delivery vehicles for targeted therapies. Overall exosomes lead a precision next-generation cancer theranostics era.

## 6 Conclusion

Exosome-based liquid biopsy stands poised to revolutionize liver cancer management, offering a non-invasive and comprehensive approach to diagnosis, prognosis, and treatment monitoring. While the field is relatively young, the potential is vast. Standardizing isolation and analysis protocols will be crucial for clinical translation, ensuring reliable and reproducible results across studies and institutions. Overcoming sensitivity limitations, potentially through advanced nanotechnology or single-molecule detection, will be essential for detecting early-stage disease and monitoring minimal residual disease. Addressing the inherent biological complexity of exosomes, through sophisticated bioinformatics analysis and multi-omics profiling, will unlock a deeper understanding of tumor heterogeneity and individual patient responses. As these challenges are addressed, exosome-based liquid biopsy will pave the way for a new era of personalized oncology, enabling earlier interventions, and ultimately, improved outcomes for patients with liver cancer.

## Availability of data and materials

Data sharing is not applicable to this article as no datasets were generated or analysed during the current study.

## Funding

There is no funding for this study.

## Ethical approval/patient consent

Not Applicable.

## Declaration of competing interest

The authors declare the following financial interests/personal relationships which may be considered as potential competing interests:

The authors declare no conflict of financial interests/personal relationships interest.
